# EFFECTIVENESS OF SMOKING CESSATION INTERVENTIONS WITH LDCT LUNG CANCER SCREENING AMONG AFRICAN AMERICAN SMOKERS

**DOI:** 10.1093/geroni/igac059.1164

**Published:** 2022-12-20

**Authors:** Tung-Sung Tseng, Yu Hsiang Kao, Mirandy Li

**Affiliations:** LSUHSC, New Orleans, Louisiana, United States; LSUHSC, New Orleans, Louisiana, United States; LSUHSC, New Orleans, Louisiana, United States

## Abstract

Low dose computed tomography (LDCT) screening can detect lung cancer early and decrease lung cancer-specific mortality for current smokers but remains under-utilized among these populations. Although African-American smokers tend to smoke less and have lower smoking pack-year histories, they have lower quit rates, higher rates of mortality from lung cancer than other racial/ethnic groups. This study examined the effectiveness of a smoking cessation intervention integrating LDCT screening among African-American smokers. This study recruited 60 African-American daily smokers over the age of 55 who qualified for LDCT screening. Participants were randomly and equally assigned into two groups (intervention and control). Overall, the mean age was 61.0 years old (standard deviation, 5.5), 61.7% of the participants were female and, 91.7% had lower incomes (<$20,000). Descriptive statistics were used to summarize demographics, smoking status, knowledge, attitudes, and stage of change for smoking cessation. The findings showed that participants in the intervention group had a lower number of daily cigarettes smoked (9.5 vs. 11.0) and a higher reduction in the number of daily cigarettes smoked (-2.3 vs. -0.9) than those in the control group. Participants in the intervention group were more likely to be in the preparation stage of the stages of change model (50.0% vs. 40.0%), progress in the stage of change (36.7% vs. 16.7%), or report already having had quit smoking (10.0% vs. 3.3%) than those in the control group. LDCT screening represents a potential "teachable moment" for African-American smokers, which may encourage them to consider these strategies for smoking cessation.

